# A Mind-Reader Does Not Always Have Deontological Moral Judgments and Prosocial Behavior: A Developmental Perspective

**DOI:** 10.3389/fpsyg.2016.01261

**Published:** 2016-08-23

**Authors:** Jian Hao, Yanchun Liu

**Affiliations:** ^1^Beijing Key Laboratory of Learning and Cognition, Department of Psychology, College of Education, Capital Normal UniversityBeijing, China; ^2^Youth Work Department, China Youth University of Political StudiesBeijing, China

**Keywords:** theory of mind, moral judgment, deontological moral judgment, prosocial behavior, development

## Abstract

The rationalistic theories of morality emphasize that reasoning plays an important role in moral judgments and prosocial behavior. Theory of mind as a reasoning ability in the mental domain has been considered a facilitator of moral development. The present study examined whether theory of mind was consistently positively associated with morality from middle childhood to late adulthood. Two hundred and four participants, including 48 elementary school children, 45 adolescents, 62 younger adults, and 49 older adults, completed theory of mind, moral judgment and prosocial behavior tasks. Theory of mind was measured with strange stories that tapped into an understanding of lies, white lies, double bluffs, irony, and persuasion. Moral judgments were measured with variants of the trolley dilemma. Prosocial behavior was measured through participants' performance in an interactive situation in which a helping request was made. The results indicated specific rather than similar developmental trajectories of theory of mind, moral judgments, and prosocial behavior. There was a quadratic trend in theory of mind, a combination of quadratic and cubic trends in deontological moral judgments and a linear decline in helping behavior. It is thus suggested that theory of mind may not be associated with morality in an unchanging way during development. Further results indicated that theory of mind and deontological moral judgments were negatively correlated for children, adolescents, and older adults but positively correlated for younger adults. Theory of mind and helping behavior were positively correlated for children but negatively correlated for adolescents. However, the relationships disappeared in adulthood. In sum, the present study reveals that theory of mind may be a nice tool for its facilitation of deontological moral judgments and prosocial behavior, but it may also be a nasty tool for its blocking of deontological moral judgments and prosocial behavior. Moreover, theory of mind may be a permanent tool for moral judgment development but a temporary tool for prosocial behavior development. Thus, the present study enriches the rationalistic theories of morality from a developmental perspective. Different relationships between theory of mind and morality from middle childhood to late adulthood are discussed.

## Introduction

A person with moral judgments and behavior is respectable. He or she can make moral judgments between right and wrong and display prosocial behavior when confronting moral situations. Individuals' moral development is important for their normal social interaction. Studies indicate that moral development is positively related to popularity and reputation within one's peer group in early developmental stages (Peterson and Siegal, 2002; Wardle et al., 2011).

Moral development may especially require mental state reasoning, i.e., theory of mind (Premack and Woodruff, 1978). For example, when an individual infers others' intentions and beliefs behind their actions, he or she is more likely to make impartial moral judgments about those actions. Additionally, understanding others' desires and emotions may facilitate prosocial behavior toward others. It is thus suggested that theory of mind helps moral development. However, Happé and Frith (1996) found that children with conduct disorder passed standard theory of mind tasks, i.e., false belief tasks but were rated as having significant antisocial behavior. Therefore, nice and nasty theory of mind are differentiated (Happé and Frith, 1996; Ronald et al., 2005). According to this theory, both specific prosocial behavior (e.g., helping behavior) and antisocial behavior (e.g., lying behavior) necessitate mental state reasoning. In other words, individuals with advanced theory of mind may not always behave prosocially.

Previous studies have examined the relationships between theory of mind and morality. With regard to moral judgments, some studies have investigated the relationship between preschoolers' theory of mind and moral judgments. Lane et al. (2010) found that preschoolers' false belief understanding positively predicted their psychological-needs reasoning in moral judgments. Smetana et al. (2012) reported that preschoolers' performance on theory of mind tasks assessing belief, desire, and belief-emotion relationship understanding had both positive and negative influences on their moral judgments on moral transgressions in terms of rule independence during different developmental intervals. Adult social neuroscience studies have confirmed that moral judgments recruited several brain regions relating to theory of mind, such as the right temporo-parietal junction (Bzdok et al., 2012; Young and Dungan, 2012). This region is responsible for reasoning about cognitive mental states such as beliefs and intentions (Young and Dungan, 2012). Higher activity in the right temporo-parietal junction has been found to be related to more lenient moral judgments regarding accidental harm (Young and Saxe, 2009). Researchers have made a distinction between cognitive and affective theory of mind (Abu-Akel and Shamay-Tsoory, 2011; Wang and Su, 2013). Cognitive theory of mind involves understanding of beliefs and intentions, whereas affective theory of mind involves understanding of emotions and feelings. According to this differentiation, previous studies indicate that understanding of cognitive mental states seems to be related to moral judgments across each developmental stage. However, the developmental changes in the relationships between understanding of cognitive mental states and moral judgments are still not clear.

Many studies have investigated the relationships between theory of mind and morals-related behavior. During preschool years, children's overall performance on cognitive and affective theory of mind tasks and their false belief understanding have been positively related to their prosocial behavior (Moore et al., 1998; Caputi et al., 2012; Yagmurlu, 2014). Using the DG and UG games, false belief understanding, cognitive perspective-taking, and emotion attribution to self have also been found to be positively associated with allocations in the games (Gummerum et al., 2010; Takagishi et al., 2010, 2014). Limited studies show that children who pass false belief tasks allocate less than those who fail (Cowell et al., 2015). When children enter elementary school, their overall performance on cognitive and affective theory of mind tasks continues to positively predict their prosocial behavior and negatively predict their bullying behavior (Caputi et al., 2012; Shakoor et al., 2012). A few studies have also indicated that children who engage in specific antisocial behavior perform as well on cognitive theory of mind tasks as prosocial children (Gasser and Keller, 2009; Lonigro et al., 2014). Limited evidence has been found for the relationships between theory of mind and morals-related behavior during adolescence and adulthood. One study indicated that intention and emotion understanding were positive predictors of preadolescents' prosocial behavior (Čavojová et al., 2011). Thus, behavioral studies suggest that understanding of both cognitive and affective mental states are related to morals-related behavior. In addition, developmental and adult social neuroscience studies have shed light on the role of cognitive processes in morals-related behavior. Studies have shown an association between children's sharing behavior and their differences in later waveforms (LPP; Cowell and Decety, 2015) and relationships between adults' behavioral altruism and their gray matter volume in the right temporo-parietal junction (Morishima et al., 2012). In sum, previous studies have indicated that theory of mind may be associated with moral judgments across development, but how they are related during different developmental stages needs to be further clarified. Furthermore, theory of mind is beneficial to prosocial behavior in preschool years. During middle childhood, theory of mind is generally positively related to prosocial behavior even if some individuals may use it to carry out antisocial behavior. The relationships between theory of mind and prosocial behavior in adolescence and adulthood are still not clear.

Therefore, it is necessary to clarify the relationships between theory of mind and morality from middle childhood to adulthood, and this clarification is theoretically important. There has been a long debate on the roles of cognitive and emotional processes in morality. Cognitive-developmental theories emphasize that reasoning and reflection play a key role in moral development (Piaget, 1965; Kohlberg, 1969). Moral judgments in Kohlberg's theory represent “underlying thought organization rather than specific responses” (Nisan and Kohlberg, 1982, p. 865), which indicates the importance of reasoning in moral judgments. The social domain theory (Turiel, 1983, 1998) also focuses on individuals' understanding and interpretations of social situations. It further makes a distinction between understanding of events in the moral and conventional domains. Moral transgressions are judged wrong regardless of explicit rules and authority (Turiel, 2008a). The above theories represent the perspectives of rationalism that were mentioned by Haidt (2012). However, the social intuitionalist model emphasizes that moral judgments are driven by quick processes of intuitions (Haidt, 2001, 2013). Moreover, emotion is considered more influential in moral actions (Haidt, 2001). Therefore, intuition and emotion play central roles in morality according to the model. Recent neuroscience work has shown that moral judgments involve the brain regions relating to theory of mind and those related to emotional processes (Bzdok et al., 2012; Young and Dungan, 2012). These findings provide neural bases for the cognitive or emotional mechanisms of morality. Thus, an association between theory of mind and morality will provide evidence for the rationalistic perspective of morality. Dissociation between them may suggest that morality depends on other mechanisms.

Several issues need to be clarified regarding the relationships between theory of mind and morality during development. First, it is not clear whether the developmental trends in theory of mind, moral judgments, and prosocial behavior are the same. Previous studies show that theory of mind increases from middle childhood to adolescence (Scheeren et al., 2013) and then declines from early to late adulthood (e.g., Henry et al., 2013). It is thus suggested that theory of mind may display an inverted U-shaped developmental trend. However, the developmental trajectories of moral judgments and prosocial behavior are not clear. According to Kohlberg's stages of moral judgments (Kohlberg, 1976), there are qualitative changes in moral judgments from childhood to adulthood. Thus, the developmental trajectory of moral judgments may be sharper than that of theory of mind. Because morals-related behavior is dissociated from moral judgments to some extent (Janssens and Deković, 1997; Derryberry and Thoma, 2005; Gasser and Malti, 2012), they may have different developmental trajectories. Therefore, it is hypothesized that there are specific developmental trends in theory of mind, moral judgments, and prosocial behavior. This hypothesis implies that theory of mind may not be associated with morality in an unchanging way during development. Second, developmental relationships between theory of mind and moral judgments still need to be clarified. According to Kohlberg's (1969) cognitive-developmental theory and findings of moral neuroscience (e.g., Blair et al., 2006; Young and Dungan, 2012), moral judgments require conscious reasoning from childhood to adulthood. Thus, it is hypothesized that theory of mind may continue to be associated with moral judgments across development. Third, it is important to further clarify the relationships between theory of mind and prosocial behavior during development. Previous studies have shown that theory of mind is generally positively related to prosocial behavior in middle childhood (e.g., Caputi et al., 2012). Thus, it is hypothesized that theory of mind and prosocial behavior are positively associated in middle childhood. However, as individuals enter adolescence, more antisocial behavior emerges. For example, indirect bullying is most common in adolescence (Vaillancourt, 2005). This form of bullying in particular requires theory of mind (Sutton et al., 1999). Thus, it is hypothesized that theory of mind may be negatively related to prosocial behavior in adolescence. As for younger and older adults, the relationships between theory of mind and prosocial behavior are seldom reported. Previous studies have shown that theory of mind develops quickly during earlier developmental stages. Children and adolescents' understanding of white lies, second-order false beliefs, double bluffs, faux pas, and sarcasm increases with age (Broomfield et al., 2002; Scheeren et al., 2013). Thus, individuals in earlier developmental stages may use theory of mind more frequently to solve moral problems than do adults. By contrast, intuition and emotion are considered more influential on adults' morality (Haidt, 2001, 2013). Therefore, it is hypothesized that the link between theory of mind and prosocial behavior may disappear in adulthood.

The present study aimed at examining the relationships between theory of mind and morality from middle childhood to late adulthood. Elementary school children, adolescents, younger adults, and older adults completed experimental tasks assessing their theory of mind, moral judgments, and prosocial behavior. Because individuals acquire basic theory of mind, such as false belief understanding, in the preschool years, an advanced test of theory of mind, strange story task (Happé, 1994) was used in the present study. This task has been proved to be suitable for both children and adults (Happé, 1994). In the present study, stories involving a lie, a double bluff, persuasion, a white lie, and irony were presented to the participants. According to previous studies, the lie, double bluff and persuasion stories involve inference of beliefs and intentions (Kemp et al., 2012; Wang and Su, 2013), and thus assess cognitive mental state understanding, while the white lie and irony stories involve reasoning about emotions and feelings (Shamay-Tsoory and Aharon-Peretz, 2007; Wang and Su, 2013), and thus measure affective mental state understanding. In addition, previous studies have revealed age-related relationships between theory of mind and morality to some extent. However, inconsistent findings also exist. Different theory of mind tasks measuring specific mental state understanding are used in previous studies, which may be the cause of the inconsistencies. Thus, it is necessary to use the strange story task measuring core components of theory of mind to further clarify whether specific mental state understanding is related to morality. Meanwhile, the use of the same theory of mind task in all age groups helps rule out the possibility that different relationships between theory of mind and morality during different developmental stages stem from different measures of theory of mind. Therefore, the strange story task measuring core components of theory of mind, i.e., belief, intention, and emotion understanding was used in the present study.

The trolley dilemma and its variants (Hauser et al., 2007) were used to assess participants' moral judgments. They involved a classic trolley dilemma (Foot, 1967; Thomson, 1985), a classic footbridge dilemma (Thomson, 1986) and two trolley dilemmas that were adapted from the classic one. In these moral dilemmas, participants were asked to decide whether it was acceptable to harm one person to save five people. There are several reasons for the use of these trolley-like dilemmas as moral judgment measures. First, the orientation of participants' moral judgments can be clearly identified. A choice to save five people indicates a utilitarian moral judgment, whereas the choice to protect the innocent person indicates a deontological moral judgment. The utilitarian principle emphasizes the maximization of the good for most people (Mill, 1998). The deontological principle emphasizes adherence to moral rules regardless of the consequences (Kant, 1959). Although individuals with utilitarian moral judgments seem to attach great importance to the good for most people, it may essentially reflect their consequence-based moral judgments. In addition, Kahane et al. (2015) noted that utilitarian moral judgments were not equal to “impartial concern for the greater good (p. 193).” Studies on psychopathy provide further evidence for the perspective. Psychopathy is characterized by severe antisocial behavior (Hare and Neumann, 2008). Individuals with more psychopathic traits prefer more utilitarian moral judgments (Glenn et al., 2010; Bartels and Pizarro, 2011; Seara-Cardoso et al., 2012). By contrast, deontological moral judgments reflect adherence to moral rules regardless of the consequences (Kant, 1959). Second, it is very important to rule out the social desirability effect, especially in moral studies. Previous studies have used some everyday situations to assess participants' moral judgments (Carlo et al., 1996; Malti et al., 2009). For example, participants were asked to decide whether to help an injured child or to go to a party (Carlo et al., 1996). It is very easy for them to give desirable answers to these familiar situations because this type of moral knowledge can be easily learned in everyday or didactic contexts. Although the trolley dilemmas are not the everyday situations that participants will encounter (Turiel, 2006, 2008b), assessing moral judgments with these dilemmas is advantageous. The advantage is that participants must make judgments without previous experience. Thus, these unfamiliar dilemmas can better assess participants' genuine moral judgments. In addition, there is an obviously desirable orientation of moral judgments in previous studies. For example, canceling a party to help an injured person is the correct choice for most people. However, in the trolley dilemmas, both choices seem reasonable. One of them is not more socially acceptable than the other. Accordingly, the social desirability effect can be better reduced. Finally, empirical studies have confirmed that both children and adults can understand the trolley-like dilemmas (Bleske-Rechek et al., 2010; Pellizzoni et al., 2010). Therefore, the trolley-like dilemmas were used to assess moral judgments in the present study. With regard to prosocial behavior, participants' helping behavior was measured through their actual interaction with the experimenter. This experimental paradigm has been administered to both children and adults (Rubin and Schneider, 1973; Gino and Desai, 2012).

There are parallels between the cognitive and behavioral measures of morality. First, both measures reflect individual differences in morality. Individuals differ in whether they make deontological moral judgments and the extent to which they make this judgment. Individuals also differ in whether they display helping behavior and the extent to which they help others. Second, the two measures reflect similar characteristics of moral development. The trolley-like dilemmas do not just involve harming or not harming others. They also involve the decision of whether to harm an innocent person even if more people will be saved. Individuals' choices of protecting the person reflect their respect for the equality of people's rights and personal dignity according to Kohlberg's (1976) theory. Similarly, individuals' prosocial behavior reflects their caring about others. Third, the opposite choice in both measures is not absolutely wrong. Thus, the trolley-like dilemmas and helping behavior experiment were used to assess moral judgments and prosocial behavior, respectively.

Several hypotheses were proposed in the present study. First, there were specific developmental trends in theory of mind, moral judgments, and prosocial behavior from middle childhood to adulthood. This hypothesis indicates, to a certain extent, that theory of mind may not be associated with morality in an unchanging way throughout development. Second, moral judgments continued to be associated with theory of mind during development. Third, theory of mind and proscial behavior were positively related in middle childhood but negatively related in adolescence. The relationships between theory of mind and prosocial behavior disappeared in adulthood. Supporting evidence for these hypotheses will help to enrich the rationalistic theories of morality from a developmental perspective.

## Methods

### Participants

A total of 204 participants were recruited from several communities in Beijing. Group 1 comprised 48 elementary school children (27 males and 21 females) ranging in age from 8.04 to 10.59 years (*M* = 9.08, *SD* = 0.67). Group 2 comprised 45 adolescents (19 males and 26 females) ranging in age from 13.14 to 15.36 years (*M* = 14.00, *SD* = 0.62). They attended junior high schools. Group 3 consisted of 62 younger adults (26 males and 36 females) ranging in age from 18.55 to 24.06 years (*M* = 20.41, *SD* = 1.07). They were undergraduate students. Group 4 were 49 older adults (25 males and 24 females) ranging in age from 60.12 to 70.13 years (*M* = 62.73, *SD* = 2.61). The older adults were asked to report their education level using the following education brackets (1 = elementary school; 2 = junior high school; 3 = senior high school; 4 = university). Eight of them have elementary school education, fourteen complete junior high school education, sixteen reach senior high school education, and ten have achieved university education. The study was approved by the Research Ethics Board of Department of Psychology of Capital Normal University. Informed written consent was obtained from all of the participants.

### Measures and procedure

#### Theory of mind

The strange stories (Happé, 1994) were used to measure participants' theory of mind. The five stories involved the understanding of a lie, a white lie, a double bluff, irony, and persuasion. Minor revisions were made based on Chinese culture. First, “Christmas” was revised to “birthday” in the white lie story. Second, the original names of the protagonists were revised to Chinese names. The participants were tested individually in a quiet room. According to Happé's (1994) procedure, each story was read aloud to the participants. Simultaneously, the story and its illustration were also presented to the participants to eliminate memory load. After reading the story, the experimenter asked the participants the test question: “Why did X (name of the protagonist) say that?” The order of the story presentation was randomized. The following is the double bluff story and the corresponding test question:

During the war, the Red army captured a member of the Blue army. They want him to tell them where his army's tanks are; they know they are either by the sea or in the mountains. They know that the prisoner will not want to tell them, he will want to save his army, and so he will certainly lie to them. The prisoner is very brave and very clever, he will not let them find his tanks. The tanks are really in the mountains. Now when the other side asks him where his tanks are, he says, “They are in the mountains” (Happé, 1994, p. 150).
Why did the prisoner say what he said? (Happé, 1994, p. 150).

According to the scoring in Happé et al. (1998), answers that fully and explicitly mentioned the correct mental states received a score of 2. Answers that partially and implicitly mentioned the correct mental states were scored 1. Incorrect answers were scored 0. Thus, the score for each story ranged from 0 to 2. The total theory of mind score ranged from 0 to 10. The answers of 20% of the participants were scored by another rater who did not know the purpose of the study. The kappa coefficients for the lie, white lie, double bluff, irony, and persuasion stories were 0.81, 0.88, 0.85, 0.82, and 0.82, respectively. Thus the inter-rater reliability was good.

#### Moral judgments

The moral dilemmas in Hauser et al. (2007) were used to assess the participants' moral judgments. There were four moral dilemmas, which were aimed at measuring the participants' utilitarian or deontological moral judgments. In these dilemmas, the participants had to decide whether to harm one person to save five people. However, some differences existed in these dilemmas. Moral dilemma 1 was the classic trolley dilemma. In this dilemma, a runaway train could be diverted to a side track so that five people on the main track could be saved. However, one person standing on the side track would be killed. Moral dilemma 2 was the classic footbridge dilemma. In this dilemma, one large person with a backpack could be pushed off a footbridge to stop a runaway train under the footbridge. Five people on the track would survive, but the large person would die. Hauser et al. (2007) noted that differences in participants' responses to the two dilemmas could be attributed to several effects. For example, they were likely to reflect the contact principle or the principle of the double effect. The contact principle emphasizes that harm with physical contact is less permissible than harm without physical contact (Cushman et al., 2006). The principle of the double effect underlines that it is less acceptable to harm a person to save more people when the harm is an intended means of saving people rather than a side effect (Kamm, 1998). Therefore, moral dilemmas 3 and 4 were designed to clarify the above possibilities. They differed only in whether harm was used as an intended means of saving five people. In moral dilemma 3, one person on a side track could be intentionally hit by a runaway train through a switch to slow down the train. As a result, five people on the main track would have time to escape, but the person on the side track would die. Moral dilemma 3 was thus termed the trolley dilemma-means. In moral dilemma 4, a heavy object on a side track could be hit by a runaway train through a switch to slow down the train. Five people on the main track would have time to escape, but one person in front of the heavy object would be killed by accident. Moral dilemma 4 was thus termed the trolley dilemma-side effect.

Minor revisions were made to the four moral dilemmas. The original names of the protagonists were changed to Chinese names. The participants were tested individually in a quiet room. Each moral story was read aloud to the participants. The story and its illustration were also presented to them. This procedure guaranteed participants' better understanding of the moral dilemmas (Hao et al., 2015). At the end of each story, the participants were asked two test questions. First, they were asked to indicate whether it was morally permissible for the protagonist to harm one person to save more people. Second, they were asked to indicate the extent to which they advocated or opposed harming the person on a rating scale from 1 (very much morally impermissible) to 6 (very much morally permissible). The order of the story presentation was randomized. Finally, participants were also asked their justifications for their choices. All the participants including the children could give reasonable justifications. Children who advocated taking action generally explained that five people would be saved. Children who opposed taking action generally explained that it was immoral to kill the lone person or the person was innocent. In addition, these trolley-like dilemmas have been successfully used in assessing moral judgments in preschool children aged 3–5 years (Pellizzoni et al., 2010) and elementary school children aged 9–10 years (Bucciarelli, 2015). It thus indicated that all the participants including the children could understand these dilemmas. The following is the trolley dilemma-classic:

Trolley dilemma-classic

Wang Dong is a passenger on a train whose driver has just shouted that the train's brakes have failed, and who then fainted of the shock. On the track ahead are five people; the banks are so steep that they will not be able to get off the track in time. The track has a side track leading off to the right, and Wang Dong can turn the train onto it. Unfortunately there is one person on the right hand track. Wang Dong can turn the train, killing the one; or she can refrain from turning the train, letting the five die (Hauser et al., 2007, p. 18).
Is it morally permissible for Wang Dong to switch the train to the side track? (Hauser et al., 2007, p. 18).
Please choose from 1 (very much morally impermissible), 2 (moderately morally impermissible), 3 (slightly morally impermissible), 4 (slightly morally permissible), 5 (moderately morally permissible), and 6 (very much morally permissible).

The deontological principle emphasizes that moral rules must be abided by regardless of the consequences (Kant, 1959). Therefore, judgments that it was morally impermissible to harm the innocent person were scored 1. The opposite judgments were scored 0. Meanwhile, the permissibility rating was reverse-scored: a higher rating indicated that the participants thought that it was more morally impermissible to harm the innocent person. In other words, the participants made deontological moral judgments to a greater extent.

#### Prosocial behavior

Gino and Desai's (2012) experimental paradigm was used to measure the participants' helping behavior. After the participants had spent some time on the theory of mind and moral judgment tasks, a helping request was made by the experimenter. The experimenter explained that another participant had been invited to take part in a survey, but the person was temporarily unavailable. The experimenter asked the participants whether they were willing to help with the extra task. The survey involved ten questions regarding decision making. The experimenter emphasized that whether to help and the number of questions with which the participant helped were completely voluntary. Thus, participants were asked two questions. The first question was “Are you willing to help me?” The question was intended to make sure that the participants understood the voluntary task. All the participants clearly expressed their willingness or unwillingness to help. If they did not want to help, the survey was skipped. If they were willing to help, they were then asked the second question “How many questions are you willing to answer?” All the volunteers answered the corresponding number of questions as they promised. Finally, the participants were debriefed. The helping behavior was scored 1, and the opposite behavior was scored 0. In addition, the number of questions with which the participants helped was recorded.

## Results

### Performance on the theory of mind, moral judgment, and prosocial behavior tasks

#### Performance on the theory of mind task

**Figure 1:**
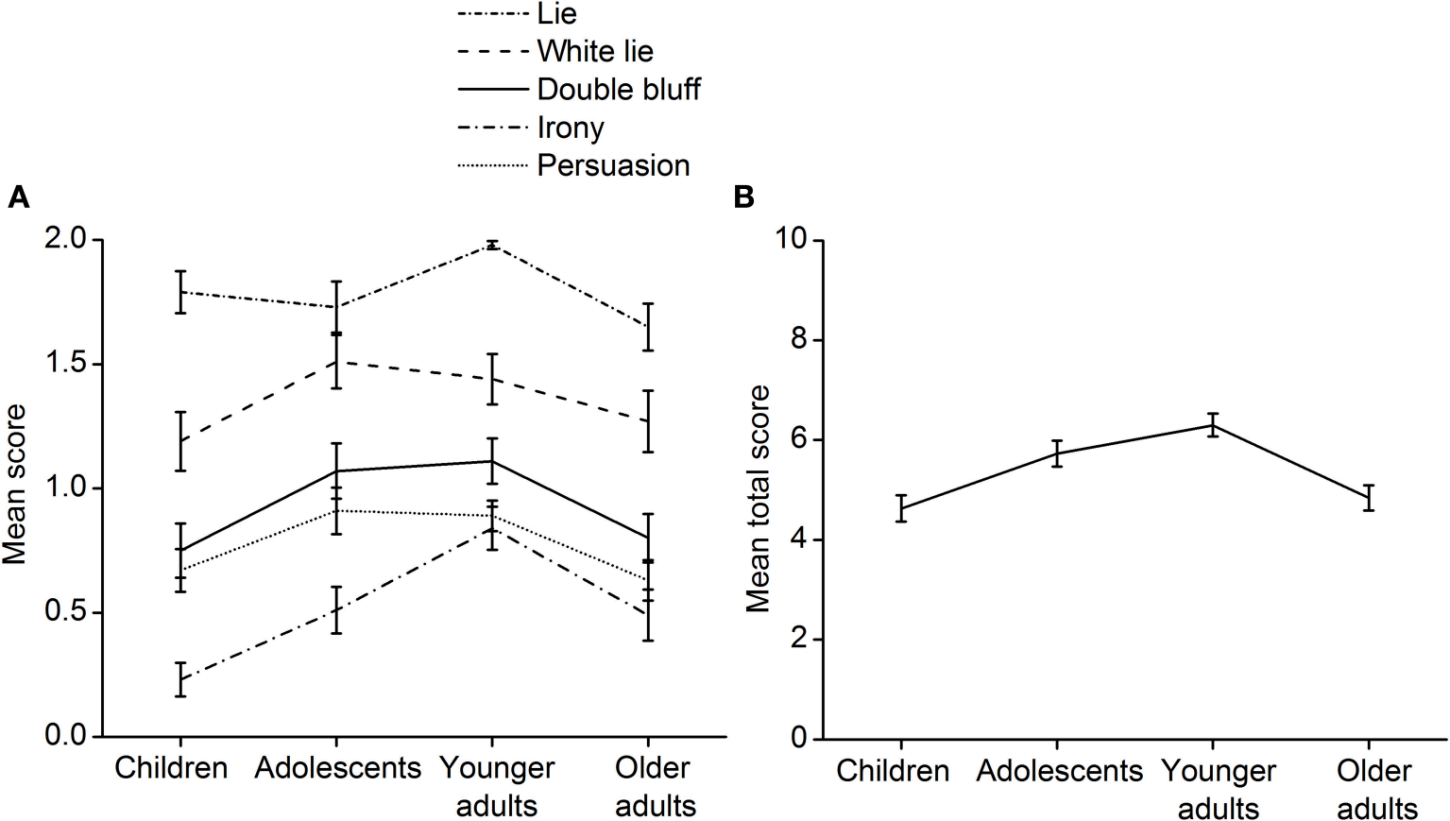
**Performance on the theory of mind task. (A)** The mean score of each age group by theory of mind story type. **(B)** The mean total theory of mind score of each age group. Error bars represent standard error.

The four age groups' performance on the theory of mind task was compared. The mean score of each age group by theory of mind story type is shown in Figure 1A. ANOVAs were conducted to examine group differences in the understanding of various mental states. There was a significant effect of age group on the lie story, *F*_(3, 199)_ = 3.74, *p* = 0.012, η^2^ = 0.053. The *post hoc* Bonferroni test found that the older adults' scores were significantly lower than the younger adults' scores, *p* = 0.011. The effect of age group failed to reach significance for the white lie story, *F*_(3, 200)_ = 1.67, *p* = 0.175, η^2^ = 0.024. For the double bluff story, the effect of age group was significant, *F*_(3, 200)_ = 3.35, *p* = 0.020, η^2^ = 0.048. The *post hoc* Bonferroni test showed that the children's scores were marginally significantly lower than those of the younger adults, *p* = 0.061. A similar age effect was found for the irony story, *F*_(3, 200)_ = 8.57, *p* < 0.001, η^2^ = 0.114. The *post hoc* Bonferroni test confirmed that the scores of the younger adults were significantly or marginally significantly higher than those of the children (*p* < 0.001), the adolescents (*p* = 0.054), and the older adults (*p* = 0.026). There was also a significant effect of age group on the persuasion story, *F*_(3, 200)_ = 3.30, *p* = 0.021, η^2^ = 0.047, but the *post hoc* Bonferroni test did not yield significant differences among the groups.

The mean total theory of mind score of each age group is shown in Figure 1B. An ANOVA indicated a significant effect of age group, *F*_(3, 199)_ = 10.41, *p* < 0.001, η^2^ = 0.136. The *post hoc* Bonferroni test demonstrated that the children's scores were significantly lower than those of the adolescents (*p* = 0.018) and the younger adults (*p* < 0.001). The adolescents' scores were marginally significantly higher than the older adults', *p* = 0.091. The younger adults outperformed the older adults, *p* < 0.001. The overall results reveal a smooth, inverted U-shaped trend in theory of mind development.

#### Performance on the moral judgment task

**Figure 2:**
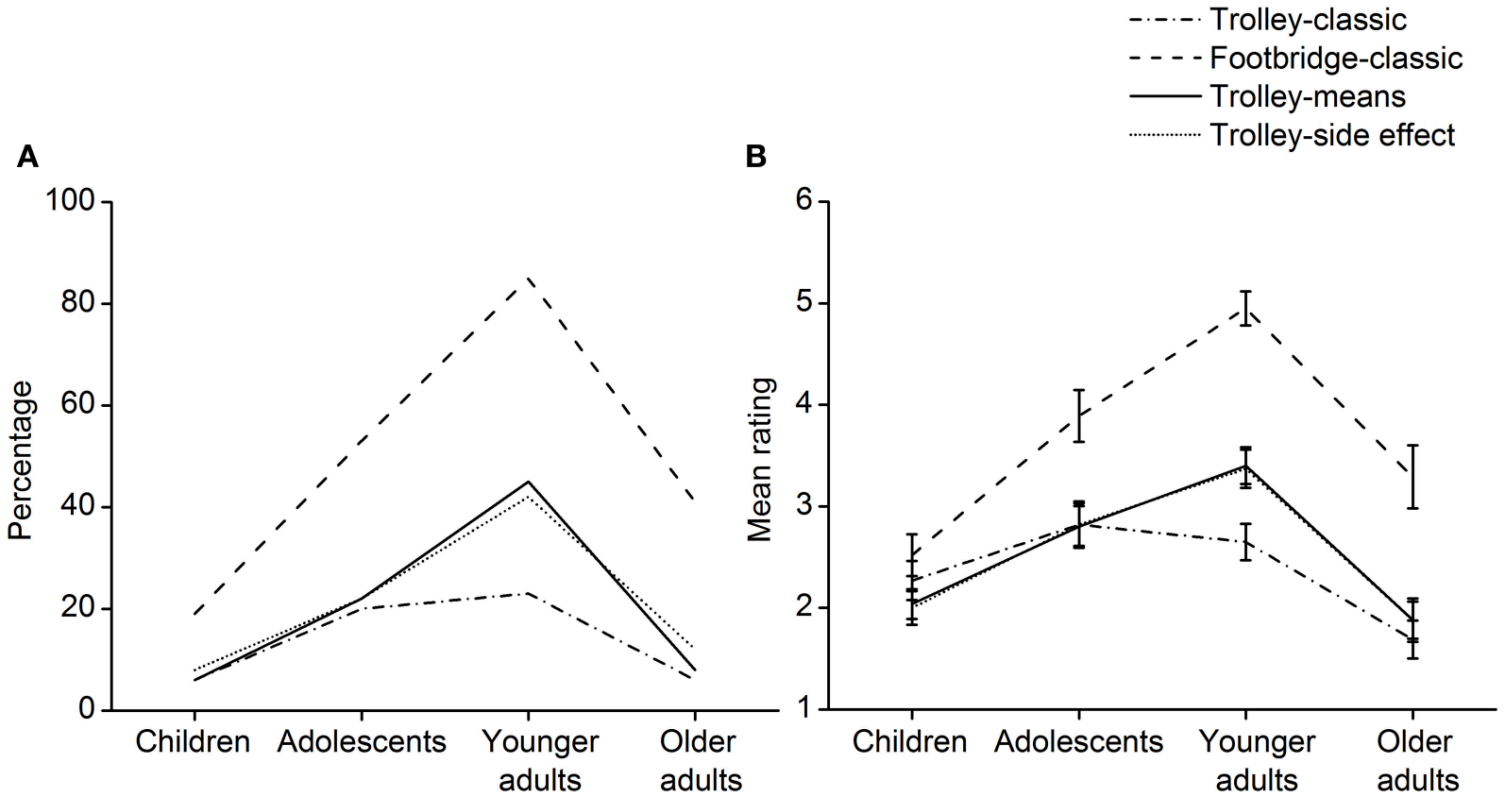
**Performance on the moral judgment task. (A)** The percentage of participants in each age group judging that it was morally impermissible to harm the innocent person by dilemma type. **(B)** The mean rating of the moral permissibility of harming the innocent person in each group by dilemma type. Higher ratings represent greater impermissibility. Error bars represent standard error.

The four age groups' performance on the moral dilemmas was analyzed. The percentage of participants in each age group judging that it was morally impermissible to harm the innocent person by dilemma type is shown in Figure 2A. Chi-square tests were performed to examine group differences in the percentage. In the trolley dilemma-classic, the percentages were significantly different among the age groups, χ${}_{\left(3,\;\text{N}\;=\;204\right)}^{2}$ = 9.92, *p* = 0.019. Higher percentages of adolescents and younger adults considered harming the innocent person morally impermissible. Group differences in the percentage were also significant for the footbridge dilemma-classic [χ${}_{\left(3,\;\text{N}\;=\;204\right)}^{2}$ = 51.59, *p* < 0.001], trolley dilemma-means [χ${}_{\left(3,\;\text{N}\;=\;204\right)}^{2}$ = 31.73, *p* < 0.001] and trolley dilemma-side effect [χ${}_{\left(3,\;\text{N}\;=\;204\right)}^{2}$ = 21.88, *p* < 0.001]. Higher percentages of younger adults made deontological moral judgments in these moral dilemmas. The percentage data show a sharp, inverted U-shaped trend in deontological moral judgment development.

In addition, the participants' performance on the trolley dilemma-classic and footbridge dilemma-classic was compared. McNemar tests indicated that in each age group, a higher percentage of participants judged that it was more morally impermissible to harm the innocent person in the footbridge dilemma-classic than in the trolley dilemma-classic (children: *p* = 0.031, adolescents: *p* < 0.001; younger adults: *p* < 0.001; older adults: *p* < 0.001). The results were probably due to the physical contact or harm as an intended means in the classic footbridge dilemma. However, the percentage of participants who judged that it was morally impermissible to harm the innocent person in the trolley dilemma-means was similar to that in the trolley dilemma-side effect for each age group (McNemar test, children: *p* = 1.000, adolescents: *p* = 1.000; younger adults: *p* = 0.754; older adults: *p* = 0.500). It is thus suggested that the different responses to the classic trolley and footbridge dilemmas in each age group are attributable to the principle of physical contact rather than the principle of double effect.

The mean rating of the moral permissibility of harming the innocent person in each age group by dilemma type is shown in Figure 2B. Higher ratings represent greater impermissibility. A 4 (age group) × 2 (trolley dilemma-classic vs. footbridge dilemma-classic) repeated measures ANOVA indicated a significant interaction effect [*F*_(3, 200)_ = 14.15, *p* < 0.001, η^2^ = 0.175], a main effect of age group [*F*_(3, 200)_ = 15.63, *p* < 0.001, η^2^ = 0.190] and a main effect of dilemma type [*F*_(1, 200)_ = 119.72, *p* < 0.001, η^2^ = 0.374]. Simple effect analysis was then performed. First, the effect of age group on permissibility rating was tested for the different dilemmas. In the trolley dilemma-classic, the effect of age group was significant, *F*_(3, 200)_ = 6.61, *p* < 0.001, η^2^ = 0.090. The *post hoc* Bonferroni test showed that compared with the older adults, the adolescents and younger adults judged that it was more impermissible to harm the innocent person, *p* = 0.001, *p* = 0.002, respectively. In the footbridge dilemma-classic, there was also a significant effect of age group, *F*_(3, 200)_ = 20.90, *p* < 0.001, η^2^ = 0.239. The *post hoc* Bonferroni test showed that the adolescents and the younger adults considered the harmful action more impermissible than the children, *p* = 0.001, *p* < 0.001, respectively. The younger adults did not accept the harmful action to a greater extent compared with the adolescents (*p* = 0.008) and the older adults (*p* < 0.001). Second, the effect of dilemma type on permissibility rating in each age group was analyzed. Participants in each age group except the children [*t*_(47)_ = 1.34, *p* = 0.188] thought that it was more impermissible to harm the innocent person in the footbridge dilemma-classic than in the trolley dilemma-classic [adolescents: *t*_(44)_ = 4.59, *p* < 0.001; younger adults: *t*_(61)_ = 11.31, *p* < 0.001; older adults: *t*_(48)_ = 5.12, *p* < 0.001]. Whether the contact principle explains the significant results for the three groups needs to be clarified by further comparing their performance on the trolley dilemma-means and trolley dilemma-side effect.

A 4 (age groups) × 2 (trolley dilemma-means vs. trolley dilemma-side effect) repeated measures ANOVA was conducted. There was no significant interaction effect, *F*_(3, 200)_ = 0.04, *p* = 0.991, η^2^ = 0.001. The absence of a main effect of dilemma type [*F*_(1, 200)_ = 0.03, *p* = 0.868, η^2^ < 0.001] meant that the participants made similar moral judgments in the two dilemmas. The main effect of age group was significant, *F*_(3, 200)_ = 17.71, *p* < 0.001, η^2^ = 0.210. The *post hoc* Bonferroni test illustrated that the harming action was judged more impermissible for the adolescents and the younger adults compared to the children (*p* = 0.015, *p* < 0.001, respectively) and the older adults (*p* = 0.002, *p* < 0.001, respectively). On the whole, the permissibility rating data also reveal a sharp, inverted U-shaped trend in deontological moral judgments from middle childhood to late adulthood. Moreover, the contact principle rather than the principle of the double effect explains the participants' different moral judgments in the classic trolley and footbridge dilemmas.

#### Performance on the prosocial behavior task

**Figure 3:**
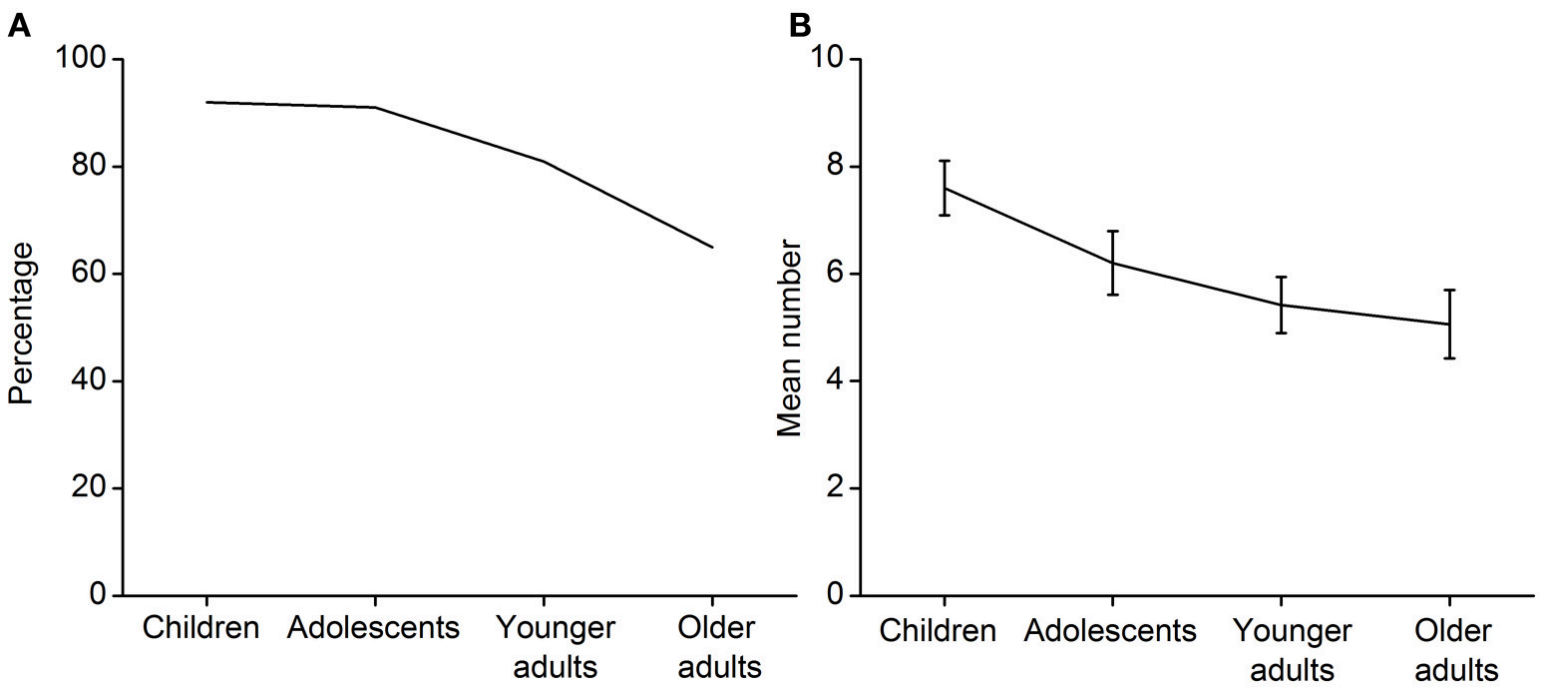
**Performance on the prosocial behavior task. (A)** The percentage of participants in each age group displaying helping behavior. **(B)** The mean number of questions with which each age group helped. Error bars represent standard error.

Performance on the helping behavior experiment in each age group was compared. The percentage of participants in each age group displaying helping behaviors is shown in Figure 3A. A chi-square test found that the percentages were significantly different among the age groups, χ${}_{\left(3,\;\text{N}\;=\;204\right)}^{2}$ = 14.81, *p* = 0.002. Higher percentages of children and adolescents provided help. The extent to which the participants helped was then analyzed. The mean number of questions with which each age group helped is shown in Figure 3B. An ANOVA indicated that there was a significant effect of age group, *F*_(3, 200)_ = 3.87, *p* = 0.010, η^2^ = 0.055. The *post hoc* Bonferroni test found that the children helped with more questions than the younger and older adults, *p* = 0.032 and *p* = 0.013, respectively. In sum, a linear decline in helping behavior seems to occur from middle childhood to late adulthood.

### Development trends in theory of mind, moral judgments, and prosocial behavior

Group difference analyses imply that there may be specific developmental trajectories in theory of mind, moral judgments, and prosocial behavior. Polynomial trend analyses were conducted to further confirm this inference. In the polynomial trend analyses, the trends that theory of mind, moral judgments, and prosocial behavior varied with age groups were tested. The trends were tested to indicate whether they followed a linear, a quadratic or a cubic trend. The results showed significant age-related quadratic trends in the understanding of white lies (contrast estimate = −0.25, *p* = 0.031), double bluffs (contrast estimate = −0.32, *p* = 0.002), and persuasion (contrast estimate = −0.25, *p* = 0.002). A significant cubic trend in the understanding of lies was found, contrast estimate = −0.20, *p* = 0.009. For the understanding of irony, the linear and quadratic trends were all significant (linear: contrast estimate = 0.25, *p* = 0.007; quadratic: contrast estimate = −0.32, *p* = 0.001). Moreover, there was a significant quadratic developmental trend in theory of mind as a whole, contrast estimate = −1.28, *p* < 0.001. In sum, the results indicate a generally quadratic trend in theory of mind development from middle childhood to late adulthood.

With regard to moral judgments, the permissibility rating data demonstrated a significant linear (contrast estimate = − 0.43, *p* = 0.030) and quadratic (contrast estimate = −0.75, *p* < 0.001) trend in deontological moral judgment development in the trolley dilemma-classic. The linear (contrast estimate = 0.75, *p* = 0.002), quadratic (contrast estimate = −1.52, *p* < 0.001), and cubic (contrast estimate = −0.54, *p* = 0.020) trends were all significant for the footbridge dilemma-classic. Deontological moral judgments in the trolley dilemma-means displayed a significant quadratic (contrast estimate = −1.14, *p* < 0.001) and cubic trend (contrast estimate = −0.44, *p* = 0.015). Significant quadratic (contrast estimate = −1.16, *p* < 0.001) and cubic (contrast estimate = −0.40, *p* = 0.047) trends were also obtained for the trolley dilemma-side effect. On the whole, the developmental trajectory of deontological moral judgments is complex, showing a combination of quadratic and cubic trends.

Finally, a significant linear decline existed in the number of questions with which participants helped, contrast estimate = −1.88, *p* = 0.001. Thus, prosocial behavior linearly declines from middle childhood to late adulthood.

### Relationships between theory of mind, moral judgments, and prosocial behavior

The trend analyses confirm that theory of mind, moral judgments, and prosocial behavior have their own specific developmental trajectories. Thus, theory of mind may not be associated with morality in an unchanging way during development. Partial correlation analyses were carried out between theory of mind and morality variables for each age group. The results are shown in Tables 1–5.

**Table 1:** **Partial correlations between theory of mind and morality variables for children**.

**Variables**	**Lie**	**White lie**	**Double bluff**	**Irony**	**Persuasion**	**Total theory of mind**
Trolley-classic 1	−0.35^*^	−0.17	−0.17	−0.12	0.14	−0.25^†^
Trolley-classic 2	−0.29^*^	−0.08	−0.25^†^	−0.01	−0.10	−0.27^†^
Footbridge-classic 1	−0.27^†^	0.15	0.02	0.01	0.15	0.04
Footbridge-classic 2	−0.40^**^	0.15	0.05	0.10	0.00	−0.10
Trolley-means 1	−0.02	−0.07	0.11	0.09	0.25^†^	0.11
Trolley-means 2	−0.22	−0.08	0.08	0.12	0.17	0.01
Trolley-side effect 1	0.01	−0.08	0.00	0.04	0.26^†^	0.06
Trolley-side effect 2	−0.16	−0.07	0.06	0.09	0.26^†^	0.05
Helping behavior 1	−0.10	0.07	0.06	0.16	0.33^*^	0.17
Helping behavior 2	−0.18	0.13	0.33^*^	0.07	0.14	0.20

Partial correlations controlling age were calculated for children. For each moral dilemma, 1 represents judgments about whether it is morally permissible to harm the innocent person (Yes = 0, No = 1); 2 represents ratings of the moral permissibility of harming the innocent person. Higher ratings represent greater impermissibility. Helping behavior 1 represents whether helping behavior is displayed (Yes = 1, No = 0). Helping behavior 2 represents the number of questions with which the participants help.

† p < 0.10,

* p < 0.05,

** p < 0.01.

**Table 2:** **Partial correlations between theory of mind and morality variables for adolescents**.

**Variables**	**Lie**	**White lie**	**Double bluff**	**Irony**	**Persuasion**	**Total theory of mind**
Trolley-classic 1	0.04	0.01	−0.14	0.04	−0.13	−0.07
Trolley-classic 2	0.13	0.02	−0.06	0.04	0.00	0.05
Footbridge-classic 1	−0.10	0.06	−0.02	0.07	−0.18	−0.06
Footbridge-classic 2	−0.03	0.07	0.00	0.12	−0.08	0.03
Trolley-means 1	−0.42^**^	−0.03	−0.14	0.09	−0.29^†^	−0.32^*^
Trolley-means 2	−0.20	0.11	−0.10	0.00	−0.20	−0.15
Trolley-side effect 1	−0.41^**^	0.12	−0.29^†^	0.27^†^	−0.29^†^	−0.25
Trolley-side effect 2	−0.21	0.27^†^	−0.20	0.22	−0.13	−0.03
Helping behavior 1	−0.12	−0.22	−0.06	−0.24	−0.03	−0.26^†^
Helping behavior 2	0.04	−0.55^***^	−0.10	−0.33^*^	−0.02	−0.38^*^

Partial correlations controlling age were calculated for adolescents.

† p < 0.10,

* p < 0.05,

** p < 0.01,

*** p < 0.001.

**Table 3:** **Partial correlations between theory of mind and morality variables for younger adults**.

**Variables**	**Lie**	**White lie**	**Double bluff**	**Irony**	**Persuasion**	**Total theory of mind**
Trolley-classic 1	0.04	−0.04	0.31^*^	−0.06	0.07	0.11
Trolley-classic 2	0.13	0.00	0.21	−0.09	0.13	0.09
Footbridge-classic 1	−0.06	−0.05	0.14	−0.10	0.00	−0.01
Footbridge-classic 2	0.09	−0.08	0.11	−0.09	0.04	−0.01
Trolley-means 1	0.11	−0.02	−0.11	−0.03	0.08	−0.04
Trolley-means 2	0.02	−0.03	−0.01	−0.05	0.12	0.00
Trolley-side effect 1	0.10	0.04	−0.01	0.01	0.08	0.04
Trolley-side effect 2	0.02	−0.01	0.02	0.03	0.10	0.04
Helping behavior 1	−0.07	0.03	0.21	0.06	−0.03	0.11
Helping behavior 2	0.14	0.04	0.08	0.10	−0.07	0.08

Partial correlations controlling age were calculated for younger adults.

* p < 0.05.

**Table 4:** **Partial correlations between theory of mind and morality variables for older adults**.

**Variables**	**Lie**	**White lie**	**Double bluff**	**Irony**	**Persuasion**	**Total theory of mind**
Trolley-classic 1	−0.14	−0.10	−0.14	0.08	0.00	−0.13
Trolley-classic 2	−0.05	−0.08	0.03	0.21	−0.08	0.01
Footbridge-classic 1	0.06	−0.16	0.06	0.22	0.02	0.06
Footbridge-classic 2	0.07	−0.07	0.05	0.16	−0.01	0.07
Trolley-means 1	−0.35^*^	−0.04	−0.07	0.02	0.05	−0.16
Trolley-means 2	−0.23	−0.07	0.05	0.01	0.05	−0.08
Trolley-side effect 1	−0.26^†^	0.00	−0.22	0.11	−0.03	−0.15
Trolley-side effect 2	−0.14	−0.10	−0.11	0.14	−0.12	−0.13
Helping behavior 1	0.09	0.12	0.23	0.00	−0.07	0.17
Helping behavior 2	0.16	0.19	0.20	0.06	0.04	0.28^†^

Partial correlations controlling age and education level were calculated for older adults.

† p < 0.10,

* p < 0.05.

**Table 5:** **Summaries of the relationships between theory of mind and morality during development**.

	**Middle childhood**	**Adolescence**	**Early adulthood**	**Late adulthood**
Theory of mind and deontological moral judgments	Negative relationships	Negative relationships	Positive relationships	Negative relationships
Theory of mind and helping behavior	Positive relationships	Negative relationships	No relationship	No relationship

The summaries are based on the significant correlations between theory of mind variables and morality variable for each age group.

There were some significant correlations between theory of mind and deontological moral judgments in each age group. The children's understanding of lies was significantly negatively correlated with whether they made deontological moral judgments in the trolley dilemma-classic (*r* = −0.35, *p* = 0.016), the degree of their deontological moral judgments in the trolley dilemma-classic (*r* = −0.29, *p* = 0.045), and footbridge dilemma-classic (*r* = −0.40, *p* = 0.006). The adolescents' understanding of lies was significantly negatively correlated with whether they made deontological moral judgments in the trolley dilemma-means (*r* = −0.42, *p* = 0.005) and trolley dilemma-side effect (*r* = −0.41, *p* = 0.006). There was also a significant negative correlation between the adolescents' total theory of mind scores and whether they made deontological moral judgments in the trolley dilemma-means, *r* = −0.32, *p* = 0.035. However, the younger adults' understanding of double bluffs was significantly positively correlated with whether they made deontological moral judgments in the trolley dilemma-classic, *r* = 0.31, *p* = 0.017. For the older adults, their understanding of lies had a significant negative correlation with whether they made deontological moral judgments in the trolley dilemma-means, *r* = −0.35, *p* = 0.018. The results indicate that individuals with better theory of mind are less likely to make deontological moral judgments in middle childhood and adolescence. In other words, they are more likely to make utilitarian moral judgments. Nevertheless, better theory of mind is related to more deontological moral judgments in early adulthood. In late adulthood, the relationships between theory of mind and moral judgments resemble those in middle childhood and adolescence. These results reveal that theory of mind and moral judgments are both positively and negatively associated during development. These relationships exist from middle childhood to late adulthood.

Some significant correlations between theory of mind and helping behavior were found in the children and adolescents. The children's understanding of persuasion was significantly positively correlated with whether they helped (*r* = 0.33, *p* = 0.026). A similar relationship was found between their understanding of double bluffs and the degree of their helping behavior (*r* = 0.33, *p* = 0.026). However, the degree of the adolescents' helping behavior was significantly negatively correlated with their total theory of mind scores (*r* = −0.38, *p* = 0.013), understanding of white lies (*r* = −0.55, *p* < 0.001), and understanding of irony (*r* = −0.33, *p* = 0.032). For the younger and older adults, there were no significant correlations between theory of mind and helping behavior. Therefore, theory of mind and prosocial behavior are both positively and negatively associated during development. Moreover, the relationships between the two disappear in adulthood.

## Discussion

Theory of mind has been considered an important facilitator of moral development. The present study enriches this view and confirms that from a developmental perspective, a mind-reader does not always have deontological moral judgments and prosocial behavior. The results found that theory of mind, moral judgments, and prosocial behavior had their own specific developmental trends. Thus, theory of mind seems not to be associated with morality in an unchanging way throughout development. Further results confirmed that theory of mind and deontological moral judgments were negatively related in middle childhood, adolescence, and late adulthood but were positively associated in early adulthood. Theory of mind and prosocial behavior were positively related in middle childhood but were negatively associated in adolescence. However, the relationships disappeared in adulthood.

The group difference and developmental trend analyses showed that theory of mind and each aspect of morality followed their own specific developmental trajectories. There was generally a quadratic developmental trend in theory of mind that presented a smooth, inverted U-shaped trajectory. Previous studies have reported a similar developmental trend, indicating that theory of mind increases from middle childhood to adolescence (Scheeren et al., 2013) and then declines from early to late adulthood (Bernstein et al., 2011; Rakoczy et al., 2012; Henry et al., 2013). Deontological moral judgments generally displayed a more complex developmental trend, which was a combination of quadratic and cubic curves. This developmental trajectory was more like a sharp, inverted U-shaped curve. Therefore, moral judgments seem to change substantially from middle childhood to late adulthood. These findings are consistent with previous theory and results. According to the cognitive-developmental theory, moral judgments mature with age, and the final postconventional level is reached in early adulthood (Colby and Kohlberg, 1987). When entering into late adulthood, individuals' moral perspective taking declines over time (Pratt et al., 1996). Moreover, the present study found that the younger adults made deontological moral judgments to a greater extent than other age groups, who were inclined to make more utilitarian moral judgments. At the postconventional level, the equality of people's rights and personal dignity is respected (Kohlberg, 1976). Younger adults might think that it was not fair to sacrifice one life to save five; thus, they chose to protect the innocent person. However, there were also some similarities among age groups in moral judgments. The overall results showed that almost all the age groups made similar moral judgments in the trolley dilemma-means and trolley dilemma-side effect but different moral judgments in the trolley dilemma-classic and footbridge dilemma-classic. As a result, the principle of physical contact rather than the principle of the double effect guided their moral judgments. Pellizzoni et al. (2010) also found the contact principle in both young children and adults. Finally, there was a linear decline in helping behavior from middle childhood to late adulthood. Previous studies have shown that sharing or donating behavior increases between the ages of 4 and 8 years (Ongley and Malti, 2014; Ongley et al., 2014) and then declines between the ages of 8 and 12 years, especially for boys (Ongley and Malti, 2014). High levels of proscoial behavior in middle childhood may be related to children's increasing abilities to decenter (Rubin and Schneider, 1973). In short, the specific developmental trajectories of theory of mind, moral judgments, and prosocial behavior imply that reasoning ability in the mental domain may not be consistently associated with morality in an unchanging way during development. There may be multiple relationships between this reasoning ability and morality.

The correlation analyses further confirmed this inference. Theory of mind and deontological moral judgments were negatively associated for children, adolescents and older adults but positively associated for younger adults. Smetana et al. (2012) also demonstrated that theory of mind had both positive and negative influences on preschoolers' moral judgments during different developmental intervals. According to Kohlberg's moral development levels, children at the preconventional level value external rules such as consequences; social systems are important for adolescents at the conventional level; and younger adults at the postconventional level begin to consider abstract principles (Nisan and Kohlberg, 1982; Colby and Kohlberg, 1987; Kohlberg, 2008). During different developmental stages, individuals' social experiences may explain their corresponding moral development levels. School experiences may make children and adolescents place greater consideration on the good for the majority of people. Wide social interaction may enable the younger adults to consider multiple perspectives of fairness rather than the apparent fairness. Therefore, there may be different perspectives on fairness for different age groups. Harming one person to save five people is fair for children and adolescents in terms of consequences and the social system, but protecting the innocent person is fair for younger adults in terms of ethical principles. Because theory of mind may give rise to inequality aversion (Fehr et al., 2008), individuals with better theory of mind are more likely to make fair choices. In the present study, children and adolescents with better theory of mind thus made less deontological judgments, whereas younger adults made more deontological judgments. As for older adults, some studies find a regression of their moral judgments (Del Vento Bielby and Papalia, 1975), but others find little change in moral judgments from middle to late adulthood (Pratt et al., 1991). When entering into the late adulthood, individuals' social interaction becomes limited. Their reasoning regarding interpersonal situations tends toward simplicity (Pratt et al., 1996). Thus, in the present study, the older adults might simply consider apparent fairness, believing that saving more people was fair despite the sacrifice of one person. In other words, they preferred less deontological judgments. The relationships between theory of mind and deontological moral judgment in them were thus negative, as was explained for children and adolescents. In addition, the present study showed that the understanding of lies and double bluffs was closely related to deontological moral judgments. These results were not by accident. Cognitive and affective theory of mind have been differentiated in previous studies (Bodden et al., 2013; Kim et al., 2016). The former involves the understanding of beliefs and intentions, and the latter involves the understanding of emotions and feelings (Wang and Su, 2013). In the present study, the lie, double bluffs, and persuasion stories focused on the inference of cognitive states, whereas the white lie and irony stories focused on the inference of emotional states. Fehr et al. (2008) proposed that individuals with better theory of mind might care more about what others thought about them. Therefore, individuals with better understanding of cognitive states may be good at inference of others' perspectives of their choice in the trolley-like dilemmas and make more acceptable moral judgments. Neuroimaging studies also indicate that inference of cognitive states such as beliefs and intentions is important for moral judgment (Young and Dungan, 2012). Furthermore, theory of mind was found to be associated with moral judgments in specific moral dilemmas for specific age groups. Because all of the age groups except the children generally thought that it was more impermissible to directly harm a person in any case, there were no relationships between theory of mind and moral judgments in the footbridge dilemma-classic in these groups. According to Piaget's (1965) stages of moral development, after the age of 10, individuals enter a stage of autonomous morality, placing greater consideration on intentions and motives. Thus, in the present study, adolescents and older adults were more sensitive to the trolley dilemma-means and trolley dilemma-side effect, which clearly involved intentional or unintentional harm. For the younger adults, the trolley dilemma-classic might implicitly induce grater consideration of intentions. For example, participants mentioned that in the dilemma it was not necessary to intentionally harm the lone person to save the five people (Hauser et al., 2007). Thus, the younger adults were more sensitive to the dilemma. In sum, the results indicate that theory of mind may be a nice tool for its facilitation of deontological moral judgments. It may also be a nasty tool for its blocking of deontological moral judgments. Moreover, theory of mind may be a permanent tool for moral judgment development.

The present study also investigated the relationships between theory of mind and prosocial behavior during development. Some studies have examined children's spontaneous prosocial behavior (Rubin and Schneider, 1973; Eisenberg-Berg and Hand, 1979). However, a number of studies have also focused on children and adolescents' response to requests to donate things or complete extra tasks (Eisenberg et al., 1987, 1991, 1995; Ongley et al., 2014). First, the paradigms of spontaneous prosocial behavior rely on behavioral observation and lack experimental control. Second, the experimenter who requested help in the present paradigm was just a common stranger rather than one of the children's important others, such as a parent or a teacher. Thus, the experimenter was not an authority figure and children did not need to obey the experimenter. Third, children were clearly told that they could skip the extra task if they were not willing to help. Therefore, the present experimental paradigm assessed genuine helping behavior. The results showed that theory of mind was positively associated with helping behavior for children but negatively related to helping behavior for adolescents. Caputi et al. (2012) indicated that theory of mind ultimately influenced peer relationships via social behavior. Children's prosocial tendencies are associated with their popularity (Deković and Gerris, 1994; Greener, 2000; Warden and Mackinnon, 2003), but specific antisocial behavior is related to adolescents' popularity (Cillessen and Borch, 2006; Mayeux, 2014; Stoltz et al., 2016). Thus, children tended to use theory of mind more prosocially, whereas adolescents tended to use it less prosocially in the present study. These results are consistent with the theory of nice and nasty theory of mind to some extent (Happé and Frith, 1996; Ronald et al., 2005). The present study also found that understanding of double bluffs, persuasion, white lies and irony was related to helping behavior. Individuals with good performance on double bluff and persuasion stories could better understand others' cognitive states and thus grasp the experimenter's intention to seek help to a greater extent. Good performance on white lie and irony stories reflected better understanding of emotional states. This would help comprehend the experimenter's anxiety about the absence of the invited participant. However, whether to behave prosocially after mental state reasoning depends on the developmental characteristics, as stated above. In addition, the results showed that the relationships between theory of mind and helping behavior disappeared in adulthood. Novakova and Flegr (2013) found that the more the amount at stake were, the less adults wanted to share in the dictator and ultimatum games. The result indicates that adults seem to be more like “homo economicus,” considering the cost of prosocial behavior. Thus, adults as homo economicus may not necessarily behave prosocially after they infer others' mental states. Their prosocial behavior may require more affective motives. Hardy (2006) has proposed that “it is moral emotion that provides the motivating “spark” that leads to moral action” (p. 208). Studies on adults are consistent with the affective mechanism, confirming the role of empathy in prosocial behavior in adulthood (Sze et al., 2012; Lockwood et al., 2014; Light et al., 2015). Therefore, the relationships between theory of mind and helping behavior were not found in adults. In sum, the results indicate that theory of mind may be a nice tool for its facilitation of prosocial behavior. However, it may also be a nasty tool for its blocking of prosocial behavior. Moreover, theory of mind may be a temporary tool for prosocial behavior development.

There are some limitations in the present study. First, the strange story task was used in the present study because it measures different aspects of theory of mind with stories of similar format. More typical stories measuring cognitive and affective theory of mind can be chosen in future studies. For example, in addition to the five stories used in the present study, second-order false belief stories can be used to assess cognitive theory of mind, whereas faux pas stories can be used to assess affective theory of mind (Wang and Su, 2013). Second, the present study focuses on judgments about right or wrong, i.e., evaluating whether it is morally permissible to treat others in specific ways. Thus, the trolley-like dilemmas were used to measure moral judgments and reduce the social desirability effect. However, the trolley-like dilemmas may not be familiar to children. Future studies focusing on reasoning behind moral judgments can use everyday moral dilemmas (e.g., Eisenberg et al., 1983) to further examine relationships between theory of mind and moral reasoning during development. Because moral reasoning relies on internal reasoning ability, the social desirability effect will be ruled out to some extent. Third, although there are some parallels between measures of moral judgments and prosocial behavior in the present study, an important distinction also exists between the two measures. Moral judgments involve evaluating what is morally permissible regarding how to treat others, whereas prosocial behavior involves a positive but not obligatory behavior. Thus, future studies can examine proscriptive behavior (Janoff-Bulman et al., 2009), such as cheating behavior, to make the cognitive and behavioral measures of morality more parallel.

In addition, a high number of correlational analyses were conducted to explore the relationships between theory of mind and moral judgments and prosocial behavior during development. Thus, the problem of inflation of type one error may exist. The alpha level was not adjusted downwards because this adjustment may be conservative for initial clarification of the trends in relationships between theory of mind and morality during development. Meanwhile, the adjustment may increase the chance of making a type two error. However, the type one error needs to be better control in future studies which aim to further confirm the relationships between key theory of mind variables and morality variables during development.

In conclusion, the present study indicates that reasoning ability in the mental domain is not always beneficial to moral development from middle childhood to late adulthood. This reasoning ability may be a nice tool for its facilitation of deontological moral judgments and prosocial behavior, but it may also be a nasty tool for its blocking of deontological moral judgments and prosocial behavior. It may be a permanent tool for moral judgment development but a temporary tool for prosocial behavior development. Thus, the present study enriches the rationalistic theories of morality from a developmental perspective.

## Author contributions

JH proposed the concept and designed the study; YL performed the acquisition of data for the study; JH performed the analysis and interpretation of the data. JH drafted the study; JH and YL revised the study for important intellectual content. All the authors finally approved the version to be published. All the authors agreed to be accountable for all aspects of the study in terms of ensuring that questions related to the accuracy or integrity of any part of the study are appropriately investigated and resolved.

### Conflict of interest statement

The authors declare that the research was conducted in the absence of any commercial or financial relationships that could be construed as a potential conflict of interest.
